# Sphingosine 1-Phosphate Receptor 1 Is Required for MMP-2 Function in Bone Marrow Mesenchymal Stromal Cells: Implications for Cytoskeleton Assembly and Proliferation

**DOI:** 10.1155/2018/5034679

**Published:** 2018-03-11

**Authors:** Chiara Sassoli, Federica Pierucci, Alessia Tani, Alessia Frati, Flaminia Chellini, Francesca Matteini, Ambra Vestri, Giulia Anderloni, Daniele Nosi, Sandra Zecchi-Orlandini, Elisabetta Meacci

**Affiliations:** ^1^Section of Anatomy and Histology, Department of Experimental and Clinical Medicine, University of Florence, 3 Largo Brambilla, 50134 Florence, Italy; ^2^Unit of Biochemical Sciences and Molecular Biology, Department of Experimental and Clinical Biomedical Sciences “Mario Serio”, University of Florence, 50 Viale GB Morgagni, 50134 Florence, Italy

## Abstract

Bone marrow-derived mesenchymal stromal cell- (BM-MSC-) based therapy is a promising option for regenerative medicine. An important role in the control of the processes influencing the BM-MSC therapeutic efficacy, namely, extracellular matrix remodelling and proliferation and secretion ability, is played by matrix metalloproteinase- (MMP-) 2. Therefore, the identification of paracrine/autocrine regulators of MMP-2 function may be of great relevance for improving BM-MSC therapeutic potential. We recently reported that BM-MSCs release the bioactive lipid sphingosine 1-phosphate (S1P) and, here, we demonstrated an impairment of MMP-2 expression/release when the S1P receptor subtype S1PR1 is blocked. Notably, active S1PR1/MMP-2 signalling is required for F-actin structure assembly (lamellipodia, microspikes, and stress fibers) and, in turn, cell proliferation. Moreover, in experimental conditions resembling the damaged/regenerating tissue microenvironment (hypoxia), S1P/S1PR1 system is also required for HIF-1*α* expression and vinculin reduction. Our findings demonstrate for the first time the trophic role of S1P/S1PR1 signalling in maintaining BM-MSCs' ability to modulate MMP-2 function, necessary for cytoskeleton reorganization and cell proliferation in both normoxia and hypoxia. Altogether, these data provide new perspectives for considering S1P/S1PR1 signalling a pharmacological target to preserve BM-MSC properties and to potentiate their beneficial potential in tissue repair.

## 1. Introduction

Mesenchymal stromal cells (MSCs) are adult stem cells, found in situ within all adult mammalian supportive stromal tissue compartments, where they play a key role in the organization and maintenance of tissue integrity and in the physiological/pathological tissue repair [1]. However, their main source remains the bone marrow, where they were originally identified [2]. Ex vivo expanded adult bone marrow-derived MSCs (BM-MSCs) have long been viewed as a potent tool for cell therapy and regenerative medicine [1, 3, 4]; their effectiveness has been proven in many preclinical in vitro and in vivo studies and clinical trials are currently ongoing [3, 5–11].

It has been observed that the efficacy of BM-MSCs in terms of tissue repair/regeneration is mainly dependent on several properties, such as the ability of these cells to (i) migrate through the extracellular matrix (ECM) where they reside, (ii) transmigrate through the endothelial cell layer and the underlying basement membrane when ex vivo expanded and systemically administered, (iii) reach the site of injury, and (iv) survive and proliferate in the damaged tissue, where they secrete a broad range of factors (secretome) with multiple beneficial effects [3, 11–14]. Tissue-specific differentiation of transplanted BM-MSCs still remains an issue of debate. For most of these biological processes, ECM represents a key factor tightly interplaying with BM-MSCs. Indeed, structural and molecular changes in ECM composition and interactions between ECM components and the cell itself can trigger intracellular signalling pathways involved in the control of different cell processes, such as cytoskeletal rearrangement, spreading, survival, proliferation, and migration [15–17]. ECM composition and remodelling depend in part on the activity of proteolytic enzymes that selectively digest individual components of the matrix, matrix metalloproteinases (MMPs), which are constitutively expressed by a wide range of cell types, including BM-MSCs, MSCs from adipose tissue and from other tissues [14, 18–21]. Studies aimed at elucidating BM-MSC-secreted molecules, as well as their downstream signalling pathways, are remarkably increasing in recent years [3, 4, 12, 13, 22]. By contrast, the factors and the molecular mechanisms possibly involved in the modulation of BM-MSC secretome in the tissue microenvironment are far from being elucidated. Therefore, the identification of modulators of BM-MSC secretome with autocrine/paracrine actions could be of great biological relevance for improving the therapeutic/beneficial potential of BM-MSCs. Among these modulatory factors, sphingosine 1-phosphate (S1P) could represent a good candidate. S1P belongs to the large family of bioactive sphingolipids, membrane-derived lipid mediators which are generated from phospholipid membrane precursors [23–25]. The major sources of S1P are represented by activated platelets and by cells subjected to a various degree of damage or stimulation by different growth factors, thus suggesting a potential role for the sphingolipid in many physiological and pathological conditions [17, 25–31]. We have recently demonstrated that BM-MSCs are also able to produce S1P through sphingosine kinase (SphK) activation and to release it similarly to many other cell types [32]. S1P is a peculiar bioactive lipid: it acts as an intracellular mediator as well as a ligand for multiple G protein-coupled membrane receptors, a class of proteins belonging to the endothelial differentiation gene (Edg) family, currently named S1PR1-5 [25, 29]. The binding of S1P to S1PR subtypes leads to their differential coupling to heterotrimeric G-proteins and downstream effector targets, thereby promoting specific cellular responses, including migration, adhesion, survival, proliferation, and gene expression in many cell types, including stem cells [17, 25–27, 31, 33–42]. Since S1PR subtypes differ in their tissue distribution, the specific effect of S1P is determined, at least in part, by the predominance of the expressed receptor subtypes [25, 29, 31]. The therapeutic options related to S1P/S1PR signalling are numerous as demonstrated by several clinical trials [31] and the recent approval by the Food and Drug Administration and the European Medicines Agency of FTY720 (Fingolimod, Gilenya, Novartis) for the treatment of relapsing multiple sclerosis [43, 44], acting as functional S1PR1 antagonist [45].

Although the multiple functions of S1P/S1PR signalling in many cell types, the involvement of S1PR subtypes on the autocrine/paracrine action of BM-MSCs remains elusive. On these bases, the aim of this study was to evaluate whether S1P/S1PR-mediated signalling could affect the ability of BM-MSCs to express and release MMP-2, the main MMP isoform/collagenase [14, 46, 47], whose expression and activity have been demonstrated to be regulated by S1P in many cell types [34, 48, 49] and, in parallel, to affect cytoskeletal organization and cell proliferation.

The experiments were conducted in cells cultured either in normoxic and hypoxic conditions in order to mimic the microenvironment occurring in a damaged/regenerating tissue.

## 2. Materials and Methods

### 2.1. Cell Culture and Treatments

Mouse bone marrow mesenchymal stromal cells (BM-MSCs) were isolated from the femura and tibiae of male C2F1 mice, expanded in vitro, characterized, and cultured as reported previously [6].^.^The cells were plated at low- (3–5,000 cells cm^−2^) and high- (15–20,000 cells cm^−2^) density confluence and treated for 48 h with specific vehicle or with the following compounds: sphingosine kinase inhibitor (iSK, 5 *μ*M, Tocris Bioscience, Bristol, UK) [30, 32, 50]; sphingosine 1-phosphate (S1P, 1 *μ*M, Calbiochem, San Diego, CA, USA, stock solution 2 mM in DMSO) S1PR1 receptor antagonist, W146 (2 *μ*M, Tocris Bioscience, stock solution 2 mM in DMSO) [51], S1PR1 receptor agonist, SEW2871 (2 *μ*M, Tocris Bioscience, stock solution 2 mM in DMSO) [52], MMP-2, and MMP-9 inhibitor, SB-3CT (5 and 10 *μ*M, Sigma, Milan, Italy, stock solution 10 mM in DMSO) [32]. DMSO less than 0.1% has been used as a vehicle.

In parallel experiments in order to mimic the hypoxic conditions occuring in damaged/regenerating tissue, the cells were cultured in a hypoxic chamber by lowering the oxygen concentration to 2% for 48 h in the absence (vehicle) or presence of W146 or SEW2871.

### 2.2. Gelatinase Assay

The MMP activity in BM-MSCs was evaluated using EnzChek® Gelatinase/Collagenase Assay Kit (Molecular Probes, Eugene, OR, USA) which provides a highly quenched, fluorescein-labeled gelatin (DQ™ gelatin), essentially as previously reported [8]. Upon proteolytic digestion, the green fluorescence of the gelatin is revealed and can be used to measure enzymatic activity. In particular, the wells of a 96-well microplate reader were coated with 25 *μ*g ml^−1^ of DQ gelatin following the manufacturer's instructions; the cells were added to the coated wells and cultured in normoxic conditions in the absence or presence of specific compounds for 24–48 h before reading the fluorescent intensity by using a multiwell scanning spectrophotometer (ELISA reader; Amersham, Pharmacia Biotech, Cambridge, UK) at a wavelength of 515 nm.

In parallel experiments, the cells were seeded onto glass coverslips previously coated with fluorescein-conjugated DQ gelatin (25 *μ*g ml^−1^), cultured as above and then observed under a confocal Leica TCS SP5 microscope (Leica Microsystems, Mannheim, Germany).

### 2.3. Reverse Transcription (RT) and Endpoint PCR Analysis

The expression levels of mRNA for S1P receptor subtypes (S1PR) in BM-MSCs at different cell density were determined by RT-PCR as reported previously [17]. In particular, total RNA was purified with TRI REAGENT (Sigma), according to the manufacturer's instructions. Concentration and purity of extracted total RNA were evaluated by measuring the absorbance at 260 and 280 nm wavelength and the absence of degradation confirmed by agarose gel electrophoresis with ethidium bromide staining. One *μ*g of total RNA from BM-MSCs was reverse-transcribed to a single-stranded cDNA using the commercially available cDNA Synthesis Kit (SuperScript® III cells Direct cDNA Synthesis Kit; Thermo Fisher Scientific, Waltham, MA, USA) according to the manufacturer's instructions. Mouse C2C12 cells, which constitutively express S1PR1, S1PR2, and S1PR3 [33], were used as positive controls (data not shown). Samples were incubated at 25°C for 5 min, at 42°C for 50 min, and then at 70°C for 5 min in a thermal cycler (Perkin Elmer, Monza, Italy).

We designed the following forward and reverse primers: S1PR1 (NM_007901), forward 5′-CCG CAA GAA CAT CTC CAA GG-3′ (710–731 bp), reverse 5′-GGC AAT GAA GAC ACT CAG GA-3′ (781–801 bp) (transcript length 91 bp); S1PR2 (NM_010333), forward 5′-CAT CGT GGT GGA GAA TCT TCT G-3′ (137–159 bp), reverse 5′-CAG GTT GCC AAG GAA CAG GTA-3′ (204–225 bp) (transcript length 88 bp); S1PR3 (NM_007901), forward 5'-CCA CCT GCA GCT TAC TGG CC-3′ (376–396 bp), reverse 5′-GGC AAT TAG CCA GCA CAT CCC-3′ (477–498) (transcript length 122 bp); S1PR4 (NM_010102.2), forward 5′-GGA CTT CTC GGT CAC TCA GC-3′(1089–1109 bp) reverse 5′-GGC TTG CTG TCA TGT TCT CA-3′ (1236–1256 bp) (transcript length 167 bp); S1PR5 (NM_053190.2), forward 5′-GGA GGG ACT CTC CTG GAT TC-3′ (1580–1600 bp), reverse 5′-TTC CTC TGT AGC CAG CCA CT-3′ (1744–1764 bp) (transcript length 184 bp); GAPDH (XR_002379299.1), forward 5′-GGT GCT GAG TAT GTC GTG GA-3′ (342–362 bp), and reverse 5′-CCT TCC ACA ATG CCA AAG TT-3′ (483–503 bp) (transcript length 161 bp).

### 2.4. Real-Time PCR

Quantitative real-time PCR was carried out using the Rotor Gene 6000 (Corbett Research, Corbett Life Science, Concorde, NSW 2137, Australia) and Syber Green reagents (Life Technologies), consisting in a specific set of primers (200 nM) and a fluorogenic internal probe. The expression of S1PR genes was quantitated in comparison with the housekeeping gene GAPDH [17]. PCR amplifications were performed on cDNA samples corresponding to a final RNA concentration of 50 ng. PCR was performed in a total volume of 25 *μ*l containing 2 × PCR Master mix (Life Technologies). Reaction conditions were as follows: 95°C for 10 min, followed by 35–40 cycles at 95°C for 15 s alternating with 52°C or 55°C or 60°C for 1 min and 72°C for 45 s. PCR amplifications were run in duplicates. Blank controls were performed in each run.

For the evaluation of S1PR4 and S1PR5 mRNA expression, double amount of cDNA (4 *μ*l) was used and 40 cycles of amplification performed. The results of the real-time PCR were determined as Ct values, where Ct was defined as the PCR threshold cycle at which amplified product was first detected. All values were normalized to the GAPDH housekeeping gene expression and ∆Ct calculated [17]. The ratio between the fold of variation of S1P receptor expression obtained from high- and low-density BM-MSCs culture is reported.

### 2.5. Confocal Laser Scanning Microscope Analysis

Immunofluorescence analyses on fixed BM-MSCs were performed essentially as reported previously [6]. BM-MSCs grown on glass coverslips were fixed with 0.5% buffered paraformaldehyde (PFA) for 10 min at room temperature. After permeabilization with cold acetone for 3 min, the fixed cells were blocked with 0.5% bovine serum albumin (BSA; Sigma) and 3% glycerol in PBS for 20 min and then incubated overnight at 4°C, with the following primary antibodies: rabbit polyclonal anti-Ki67 (1 : 100; Abcam, Cambridge, UK), rabbit polyclonal anti-MMP-2 (1 : 200; Abcam), rabbit polyclonal anti-cortactin (1 : 50; Santa Cruz Biotechnology, Santa Cruz, CA, USA), rabbit polyclonal anti hypoxia-inducible factor-1*α* (HIF-1*α*; 1 : 100; Santa Cruz Biotechnology), and mouse monoclonal anti-vinculin (1 : 100; Sigma).

The immunoreactions were revealed by anti-rabbit Alexa Fluor 488-conjugated IgG (1 : 200; Molecular Probes) or anti-mouse Cy5-conjugated IgG. Actin filament organization was evaluated by labeling the cells with Alexa 568-labelled phalloidin (1 : 100; Molecular Probes). In some experiments, nuclei were counterstained with propidium iodide (PI, 1 : 30; Molecular Probes). Negative controls were carried out by replacing the primary antibodies with non-immune serum; cross-reactivity of the secondary antibodies was tested in control experiments in which primary antibodies were omitted. The coverslips containing the immunolabelled cells were observed under a confocal Leica TCS SP5 microscope (Leica Microsystems) equipped with a HeNe/Ar laser source for fluorescence measurements and with differential interference contrast (DIC) optics. Observations were performed using a Leica Plan Apo 63X/1.43NA oil immersion objective. Series of optical sections (1024 × 1024 pixels each; pixel size 204.3 nm) 0.4 *μ*m in thickness were taken through the depth of the cells at intervals of 0.4 *μ*m. Images were then projected onto a single “extended focus” image. Ki67 positive nuclei were evaluated in 10 random 200 × 200 *μ*m^2^ microscopic fields (63 × objective) in each cell preparation and expressed as percentage of the total cell nuclei.

Densitometric analyses of the intensity of MMP-2, HIF-1*α*, and vinculin fluorescent signals were performed on digitized images using ImageJ software (http://rsbweb.nih.gov/ij) in 20 regions of interest (ROI) of 100 *μ*m^2^ for each confocal stacks (at least 10). Colocalization analysis of cortactin and F-actin fluorescent signals was performed using ImageJ JACOP plugin [53] and the colocalization parameter, overlap coefficient, was reported.

### 2.6. Gelatin Zymography

MMP-2/collagenases activity was assessed by gelatin zymography by using conditioned medium obtained from BM-MSCs essentially as previously reported [17]. After 48 h of cell culture, the conditioned medium of BM-MSCs treated with W146 or SEW2871 or vehicle was collected and centrifuged at 10,000*g* for 15 min and stored at −20°C. Samples, 20–25 *μ*l each, were mixed with sample buffer and separated on 10% SDS-polyacrylamide gels containing gelatin (1 mg ml^−1^), and the gel was developed. The MMPs appear as bright bands within the stained gel corresponding to the position of active MMP-2.

### 2.7. Western Blotting Analysis

Cells were lysed in hypotonic medium (about 23 mM compared with 137 mM in isotonic medium) using a Dounce homogenizer, and nuclear fraction was obtained by centrifugation at 1,000*g* at 4°C. Proteins (20–30 *μ*g) from lysates were subjected to electrophoresis (SDS-PAGE) and Western blotting analysis was performed as previously described [17, 30, 32]. To immunodetect endogenous HIF, rabbit polyclonal antibody against HIF-1*α* (1 : 1000; 132 KDa; Santa Cruz Biotechnology) was utilized. Rabbit polyclonal antibody anti-Bax (1 : 500; 21 KDa; Santa Cruz Biotechnology) and rabbit polyclonal antibody anti-Beclin (1 : 500; 52 KDa; Cell Signalling Technology, Danvers, MA, USA) were used to detect the proapoptotic and the autophagy markers, respectively. Bound antibodies were revealed by anti-rabbit immunoglobulin G1 conjugated to horseradish peroxidase (Santa Cruz Biotechnology) and ECL reagents (Amersham Pharmacia Biotech, Italy). Anti *β*-actin (1 : 10,000; 42 KDa; Santa Cruz Biotechnology) was used to demonstrate the quality and equivalent loading of protein.

### 2.8. Cell Proliferation and Viability Analyses

BM-MSCs were incubated for 24 h in DMEM containing 10% FBS (Sigma) in the absence or in the presence of compounds as reported in the indicated experiments. Cells were counted after fixation and propidium iodide staining by TALI® cytometry (Life Technologies). Cell proliferation was evaluated also by Ki67 confocal immunofluorescence analysis as previously reported [32]. Cell viability was evaluated by nonradioactive cell assay (MTT) (CellTiter 96® Assay; Promega Corporation, Madison, WI, USA), according to the manufacturer's protocol and as reported previously [30, 32].

### 2.9. Statistical Analysis

Data were reported as mean ± S.E.M. Statistical significance was determined by one-way ANOVA and Newman-Keuls multiple comparison test or Student's *t*-test. A *p* value ≤ 0.05 was considered significant. Calculations were performed using GraphPad Prism software (GraphPad, San Diego, CA, USA).

## 3. Results

### 3.1. BM-MSC Gelatinolytic Activity Is Regulated by S1P/S1PR1 Axis

**Figure 1:**
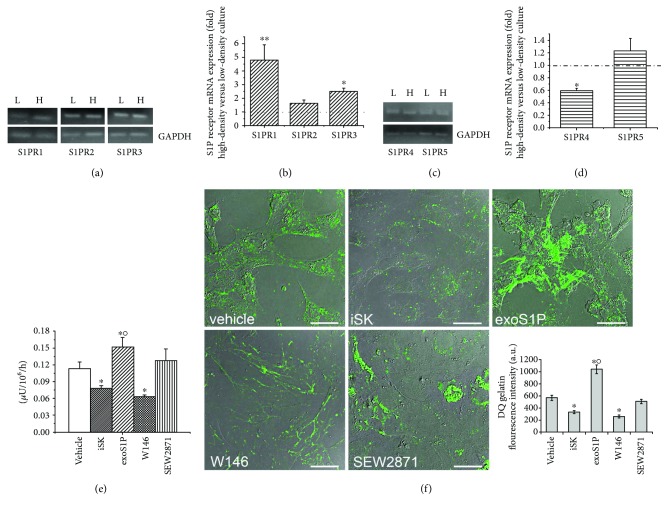
S1P receptor subtype expression and SphK/S1PR axis role in cell gelatinolytic activity. (a and c) Expression of S1P receptors by reverse transcription (RT) and real-time PCR analysis. mRNA were determined by RT of total RNA (1 *μ*g) obtained from BM-MSCs at low- (L-) and high- (H-) density culture and 2 *μ*l of cDNA (for S1PR1, S1PR2, and S1PR3 detection) or 4 *μ*l of cDNA (for S1PR4 and S1PR5 detection) were amplified as described in Section 2. Representative agarose gels of amplified DNA are shown. GAPDH amplification was used for data normalization. (b and d) Quantification of mRNA expression by real-time PCR analysis. Data are reported as mean ± S.E.M. of the ratio between the fold of variation of S1P receptor expression obtained from high- and low-density BM-MSCs culture. (e and f) BM-MSCs seeded onto fluorescein-labeled gelatin substrate- (DQ gelatin-) coated plastic culture plates (e) or glass coverslips (f) were cultured for 48 h in absence (vehicle) or in presence of the following compounds: 5 *μ*M sphingosine kinase inhibitor (iSK), 1 *μ*M exogenous sphingosine-1-phosphate (exoS1P), 2 *μ*M S1PR1 receptor antagonist, W146, and 2 *μ*M S1PR1 receptor agonist, SEW2871. (e) Spectrophotometrical quantification of the DQ gelatin fluorescence intensity revealed after proteolytic digestion of the gelatin by MMP gelatinases. (f) Representative superimposed DIC (grey) and fluorescent confocal microscopy images (green; gelatin fluorescence intensity) of fixed cells. Scale bar 30 *μ*m. Histogram shows the densitometric analysis of the intensity of the gelatin fluorescence signals performed on digitized images. Data reported as mean ± S.E.M. are representative of at least three independent experiments with similar results. Significance of differences in (b) and (d) (Student's *t*-test), ^∗^*p* < 0.05 and ^∗∗^*p* < 0.01; in (e) and (f) (one-way ANOVA and Newman-Keuls multiple comparison test), ^∗^*p* < 0.05 versus vehicle, °*p* < 0.05 versus SEW2871.

Owing to previous data demonstrating that BM-MSCs produce and release S1P [32], we evaluated the ability of the bioactive lipid to exert an autocrine action through the activation of S1P receptor-mediated signalling. First, we analysed by reverse transcription and real-time PCR the expression of the five S1P receptor (S1PR) subtypes and we found that BM-MSCs expressed three of five S1PRs, S1PR1, S1PR2, and S1PR3 (Figures 1(a) and 1(b)), whereas in this experimental conditions, S1PR4 and S1PR5 subtypes were not detectable. However, bands corresponding to S1PR4 and S1PR5 were detected when the real-time PCR amplification was performed by using double amount of cDNA (Figures 1(c) and 1(d)).

Next, we compared the expression profile of these S1PR subtypes in low-density and high-density BM-MSC cultures. This is because changes in the cell behavior occur when MSCs, recruited to the injury site in pathological conditions, start accumulating in the damaged area. Indeed, from scattered cells, they become a conspicuous pool of cells. As shown in (Figures 1(a) and 1(b)), in high-density cell culture conditions, the real-time PCR analysis indicated that S1PR1 displayed a marked increase, S1PR3 a slight one, whereas S1PR2 did not change, suggesting a prevalent role of S1PR1 in the high-density pool of BM-MSCs.

Based on these data, we focused our further investigations on the involvement of S1PR1 on the control of the secretion and activity of MMPs in high-density cell culture. Cells were first treated with a selective SphK inhibitor (iSK, 5 *μ*M), that blocks S1P synthesis, in order to evaluate the contribution of S1P inside-out signalling or with exogenous S1P (exoS1P, 1 *μ*M).

By the fluorescent gelatin degradation assay, we found that the ability of BM-MSCs to synthesize functional gelatinases was remarkably reduced when the cells were cultured in the presence of iSK (Figures 1(e) and 1(f)), indicating that S1P production is required for this function. Moreover, gelatin degradation appears to be slightly increased after stimulation with exoS1P (Figures 1 and 1(f)), further confirming that S1P/S1PR-mediated signalling plays a role in the autocrine control of MMP functionality in BM-MSCs. Moreover, we found that the cells in the presence of S1PR1 receptor antagonist, W146 (2 *μ*M), dramatically reduced their cell gelatinolytic activity (Figures 1(e) and 1(f)), without showing any particular morphological features of cell suffering, as indicated in DIC images (Figure 1(f)).

The addition of SEW2871 (2 *μ*M), a specific S1PR1 agonist, did not promote any effect as compared to control (Figures 1(e) and 1(f)), thus suggesting a potential constitutive activation of S1PR1-signalling by S1P physiologically produced inside the cells and released into the medium.

Interestingly, these data point out that S1P/S1PR1 system is required for maintaining the gelatinolytic ability of BM-MSCs.

### 3.2. S1PR1 Is Required for MMP-2 Expression/Activity in BM-MSCs

**Figure 2:**
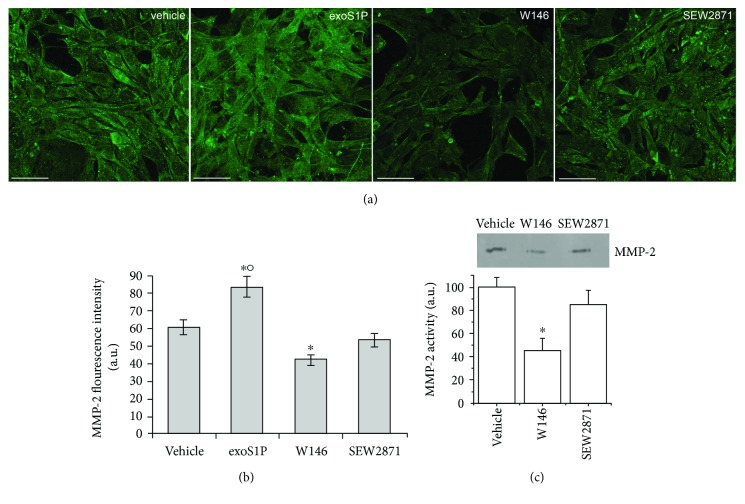
MMP-2 expression and activity. BM-MSCs were cultured for 48 h in the absence (vehicle) or in presence of 1 *μ*M exogenous sphingosine-1-phosphate (exoS1P) or 2 *μ*M S1PR1 receptor antagonist, W146, or 2 *μ*M S1PR1 receptor agonist, SEW2871. (a) Representative immunofluorescence confocal images of fixed cells on glass coverslips immunostained with antibodies against MMP-2 (green). Scale bar 50 *μ*m. The images are representative of at least three independent experiments with similar results. (b) Densitometric analysis of the intensity of the MMP-2 fluorescence signal performed on digitized images. (c) Zymography. A representative gelatin zymography of MMP-2 from conditioned media obtained from BM-MSCs incubated in absence (vehicle) or in presence of W146 or SEW2871 for 48 h. Densitometry scanning from at least three separate experiments was performed and data, expressed as relative OD values (a.u.) to those of control group (vehicle) set to 100, are reported in the histogram. Data are mean ± S.E.M. Significance of difference in (b) (one-way ANOVA and Newman-Keuls multiple comparison tests), ^∗^*p* < 0.05 versus vehicle, °*p* < 0.05 versus SEW2871; in (c) (Student's *t*-test), ^∗^*p* < 0.05 versus vehicle.

Successively, we examined the role of S1P/S1PR1 axis in the control of MMP-2 expression/activity in BM-MSCs. According to the results of gelatin degradation assay, confocal immunofluorescence analysis revealed that the cells cultured in the presence of exoS1P exhibited a significant increase of MMP-2 expression, as compared to control cells (Figures 2(a) and 2(b)). W146 promoted a robust downregulation of the enzyme expression (Figures 2(a) and 2(b)), associated with a reduction of the release of MMP-2 active form, as judged by zymography (Figure 2(c)), further underlying the role of S1PR1-mediated signalling in the control of ECM remodelling. The treatment of the cells with SEW2871 was not able to modify MMP-2 expression (Figures 2(a) and 2(b)) and activity (Figure 2(c)) as compared to control.

### 3.3. S1PR1/MMP-2 System Affects Plasma Membrane-Associated F-Actin Structures and Cell Proliferation in BM-MSCs

**Figure 3:**
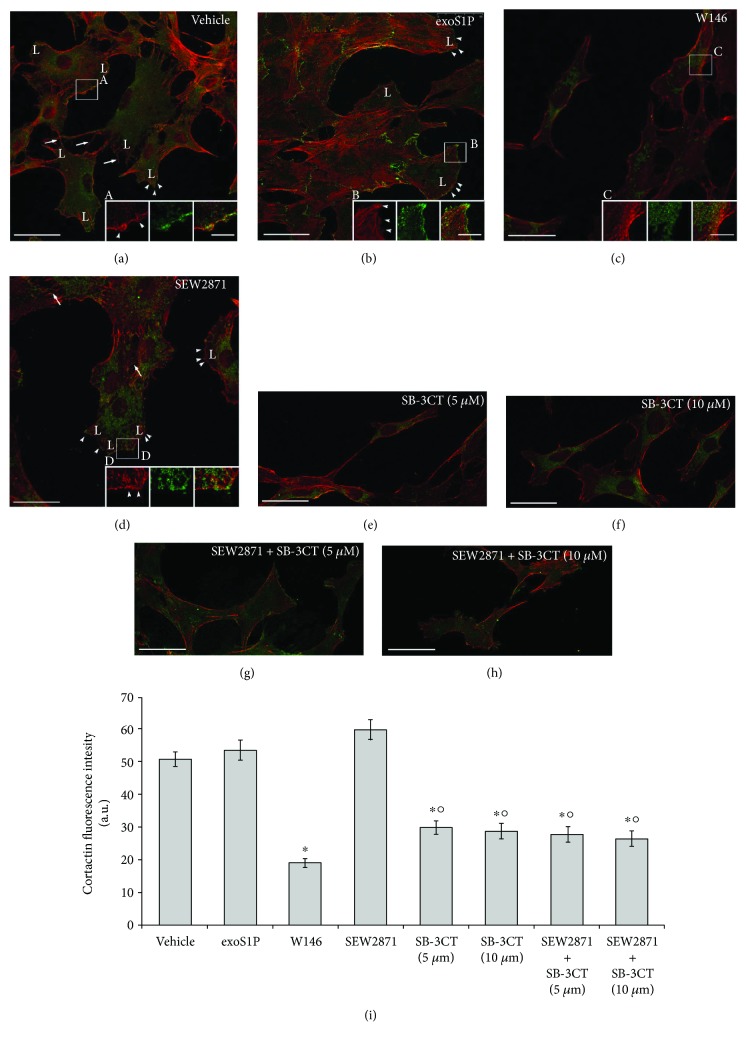
Cytoskeleton organization and cortactin expression. BM-MSCs were cultured for 48 h in the absence (vehicle) or in presence of the following compounds: 1 *μ*M exogenous sphingosine-1-phosphate (exoS1P), 2 *μ*M S1PR1 receptor antagonist, W146, and 2 *μ*M S1PR1 receptor agonist, SEW2871 and/or MMP-2/9 inhibitor, SB-3CT (5 *μ*M or 10 *μ*M). (a–h) Representative immunofluorescence confocal images of cells cultured on glass coverslips in the indicated experimental conditions, fixed and stained with Alexa 568-phalloidin to visualize actin filaments (red) and immunostained with antibodies against cortactin (green). Scale bar 50 *μ*m. Arrows indicate filopodia and arrowheads indicate lamellipodia (L). (A–D) Magnifications of the indicated squared regions of interest showing the red and green fluorescence signals separately and together. Yellow-orange colour indicates colocalization between the two fluorescence signals. Scale bar 12 *μ*m. The images are representative of at least three independent experiments with similar results. (i) Densitometric analysis of the intensity of the cortactin fluorescence signal performed on digitized images. Data are mean ± S.E.M. Significance of differences (one-way ANOVA and Newman-Keuls multiple comparison test): ^∗^*p* < 0.05 versus vehicle, °*p* < 0.05 versus SEW2871.

It has been demonstrated that MMPs are required for actin filament polymerization (F-actin) and cytoskeleton assembly in many cell types [8]. Thus, we analysed the actin cytoskeleton organization in BM-MSCs treated with exoS1P and S1PR1 ligands. The observation at confocal fluorescence microscope revealed that control cells have a polygonal appearance with actin filaments parallelly arranged, principally at the cell periphery, consistent with lamellipodia-like structures. Some cells also displayed actin-rich plasma membrane protrusions, such as filopodia and small punctate structures (microspikes) (Figure 3(a)). Moreover, the confocal immunofluorescence analysis of the expression of cortactin, a multidomain protein known to contribute to the formation of dynamic cortical actin-associated structures (such as lamellipodia) as well as to the transduction of specific cell signalling [54–56], revealed that this protein was diffused throughout the cytoplasm and, in some cells, colocalized with cortical F-actin at the cell periphery (Figures 3(a) and 3(i); overlap coefficient *r*: 0.924 ± 0.055). The cells treated with exoS1P showed actin-rich plasma membrane protrusions concomitantly with a robust increase of cytoskeleton assembly as compared to control cells, consisting in the formation of well-structured F-actin filaments spanning through the length of the cell (Figure 3(b)) according to previous reports [35] and cortactin appeared mainly cortically localized (Figures 3(b) and 3(i)). In these cells, no differences in cortactin expression and colocalization degree with F-actin (overlap coefficient *r*: 0.817 ± 0.11, *p* > 0.05 versus vehicle) were found as compared to control cells.

Of note, W146-treated cells showed a more elongated shape, a marked reduction of F-actin assembly and the disappearance of the membrane protrusions associated with a reduction of cortactin expression as compared to control cells (Figures 3(c) and 3(i); overlap coefficient *r*: 0.922 ± 0.054, *p* > 0.05 versus vehicle). The F-actin assembly and cell shape in BM-MSCs treated with SEW2871 were comparable to those of control cells (Figure 3(d)) as well as cortactin expression/distribution (Figures 3(d) and 3(i); overlap coefficient *r*: 0.923 ± 0.06, *p* > 0.05 versus vehicle).

To further evaluate the involvement of MMP-2 on the cytoskeletal remodelling triggered by S1PR1 activation, we analysed F-actin assembly and cortactin expression of the cells treated with a specific inhibitor of MMP-2/9 and SB-3CT (5 and 10 *μ*M) in absence or presence of SEW2871. We found that BM-MSCs treated with SB-3CT alone (Figures 3(e), 3(f), and 3(i)) showed a cytoskeletal disassembly, peripheral cortactin expression reduction, and a morphology similar to those of cells treated with W146 (Figures 3(c) and 3(i)). The effects elicited by the combined treatment with SB-3CT and SEW2871 (Figures 3(g)–3(i)) were not different from those induced by SB-3CT alone (Figures 3(e), 3(f), and 3(i)).

Altogether, these data suggest that the activation of MMP-2 through S1PR1-mediated signalling is required for plasma membrane-associated F-actin structures in BM-MSCs.

**Figure 4:**
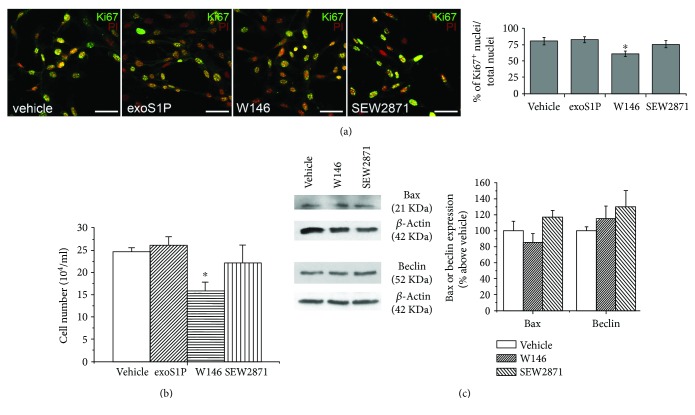
Cell proliferation and toxicity. BM-MSCs were incubated in growth medium for 24 h in absence (vehicle) or in presence of 1 *μ*M exogenous sphingosine-1-phosphate (exoS1P), 2 *μ*M S1PR1 receptor antagonist, W146, or 2 *μ*M S1PR1 receptor agonist, SEW2871. (a) Representative confocal immunofluorescence images of Ki67 expression. BM-MSCs were immunostained with the specific antibody Ki67 (green), a nuclear proliferation marker, and counterstained with propidium iodide (PI; red). Yellow colour indicates colocalization of red and green fluorescence signals. Scale bar 50 *μ*m. The images are representative of at least three independent experiments with similar results. Histogram represents quantitative analysis of Ki67 positive BM-MSC cell nuclei expressed as percentage of the total nuclei number. Data are mean ± S.E.M. (b) Cell proliferation analysis by cell counting. Synchronized BM-MSCs were collected and counted as reported in Section 2. Data are mean ± S.E.M. of four independent experiments performed in quadruplicate. (c) Western blotting analysis of apoptotic (Bax) and autophagic (Beclin) markers. Cell lysates (10–25 *μ*g) obtained from BM-MSCs were loaded onto SDS-PAGE and proteins immunodetected by specific antibodies. *β*-Actin was used as loading control. Blot shown is representative of at least three independent experiments with similar results. Data resulting from densitometric analysis of at least three independent experiments are shown in the graph (mean ± S.E.M.). Significance of differences in (a) and (b) (one-way ANOVA and Newman-Keuls multiple comparison test): ^∗^*p* < 0.05 versus vehicle.

It has been reported that plasma membrane-associated F-actin structure formation can affect cell proliferation [57–60]. Thus, we evaluated the involvement of S1PR1-triggered signalling on BM-MSCs in this biological process. The results indicate that the expression of nuclear Ki67, a specific marker of cell division, appeared reduced by approximately 30% ratio in the presence of W146 with respect to control after 24 h culture, whereas the treatment with SEW2871 was not effective (Figure 4(a)). Similar data were also obtained when proliferation was assessed by cell counting (Figure 4(b)). Notably, the reduced cell proliferation observed in the presence of W146 was not due to cell toxicity. In fact, the levels of the expression of the proapoptotic marker, Bax, and of the autophagic marker, Beclin, were no different in the presence of S1PR1 antagonist with respect to control or SEW2871 (Figure 4(c)).

Finally, the treatment with exoS1P did not affect cell proliferation consistently with cytoskeleton assembly observed in S1P-stimulated cells (Figures 4(a) and 4(b)), and suggesting that S1PR1 and the other S1PR subtypes, activated by exoS1P, are differently involved in the modulation of BM-MSC responses (namely, ECM remodelling, actin assembly, and cell proliferation).

### 3.4. S1PR1 Affects Stress Fiber Formation in BM-MSCs Cultured in Hypoxic Conditions

**Figure 5:**
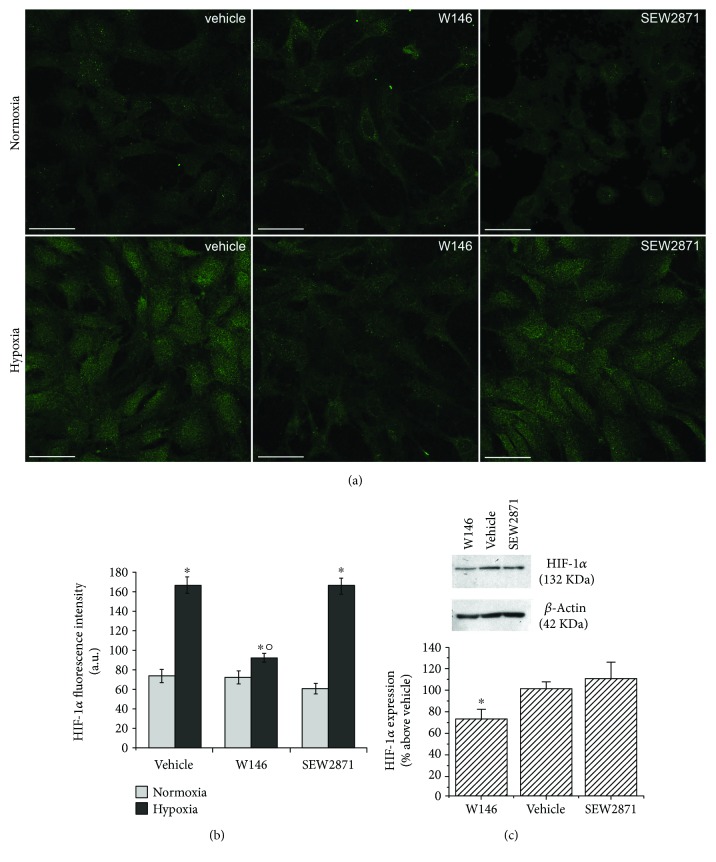
HIF-1*α* expression. BM-MSCs were cultured for 48 h in normoxic or in hypoxic conditions in the absence (vehicle) or in presence of 2 *μ*M S1PR1 receptor antagonist, W146, or 2 *μ*M S1PR1 receptor agonist, SEW2871. (a) Representative immunofluorescence confocal images of cells cultured on glass coverslips in the indicated experimental conditions, fixed and immunostained with antibodies against HIF-1*α* (green). Scale bar 50 *μ*m. The images are representative of at least three independent experiments with similar results. (b) Densitometric analysis of the intensity of the HIF-1*α* fluorescence signal performed on digitized images. Data are mean ± S.E.M. (c) Western blotting analysis of HIF-1*α*. Cell lysate (30 *μ*g) obtained from BM-MSCs cultured in hypoxia and treated as reported above was loaded onto SDS-PAGE and protein immunodetected by a specific antibody. *β*-Actin was used as loading control. Blot shown is representative of at least three independent experiments with similar results. Data resulting from densitometric analysis of at least three independent experiments is shown in the graph (mean ± S.E.M.). Significance of differences in (b) (one-way ANOVA and Newman-Keuls multiple comparison test), ^∗^*p* < 0.05 versus normoxia, °*p* < 0.05 versus vehicle hypoxia; in (c) (Student's *t*-test), ^∗^*p* < 0.05 versus vehicle.

In order to mimic the hypoxic microenvironment occurring in a damaged/regenerating tissue, parallel experiments were performed by culturing BM-MSCs in low oxygen conditions (2%). First, we evaluated by confocal immunofluorescence and Western blotting analyses the expression of HIF-1*α*, the oxygen-regulated subunit of the heterodimeric transcription factor HIF, which directs the transcriptional responses to hypoxia [61]. As expected, we found that BM-MSCs showed a significantly higher expression of HIF-1*α*, either in the cytoplasm and in the nucleus, as compared to the control cells cultured under normoxia (Figures 5(a) and 5(b)). In normoxia, HIF-1*α* was barely detected in all experimental conditions and no differences were observed among control, W146- and SEW2871-treated cells. Similarly, Western blotting analysis showed that the expression level of HIF-1*α* in W146-treated cells under hypoxia was significantly reduced of about 30%, whereas in SEW2871-treated cells, the protein content was similar to that of control cells (Figure 5(c)).

These findings are the first evidence that S1PR1-mediated signalling plays a crucial role in the control of HIF-1*α* expression in BM-MSCs under hypoxia.

**Figure 6:**
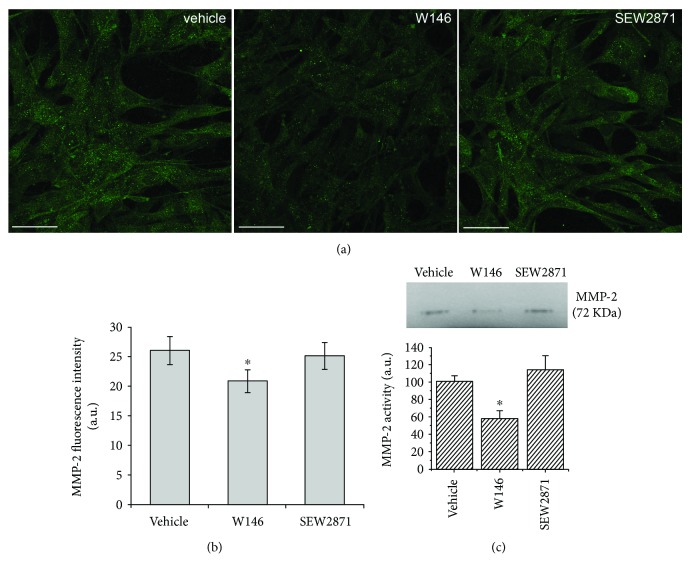
MMP-2 expression and activity in MSCs cultured under hypoxic conditions. BM-MSCs were cultured for 48 h under hypoxic conditions in the absence (vehicle) or in presence of 2 *μ*M S1PR1 receptor antagonist, W146, or 2 *μ*M S1PR1 receptor agonist, SEW2871. (a) Representative immunofluorescence confocal images of cells cultured on glass coverslips, fixed and immunostained with antibodies against MMP-2 (green). Scale bar 50 *μ*m. The images are representative of at least three independent experiments with similar results. (b) Densitometric analysis of the intensity of the MMP-2 fluorescence signal performed on digitized images. Data are mean ± S.E.M. (c) Zymography. A representative gelatin zymography of MMP-2 from conditioned media obtained from BM-MSCs incubated in the absence (vehicle) or in the presence of 2 *μ*M S1PR1 receptor antagonist, W146, or 2 *μ*M S1PR1 receptor agonist, SEW2871, for 48 h. Densitometry scanning from at least three separate experiments was performed and data, expressed as relative OD values (a.u.) to those of the control group (vehicle) set to 100, are reported in the histogram. Data are mean ± S.E.M. Significance of differences in (b) and (c) (Student's *t*-test): ^∗^*p* < 0.05 versus vehicle.

As judged by confocal immunofluorescence analysis and zymography, the cells under hypoxia showed reduced MMP-2 expression (Figures 6(a) and 6(b)) and activity (Figure 6(c)) as compared to normoxia (Figure 2) and W146 treatment, differently to SEW2871, further decreased MMP-2 expression. These findings suggest that MMP-2 expression/activity is negatively affected by hypoxic conditions in BM-MSCs.

**Figure 7:**
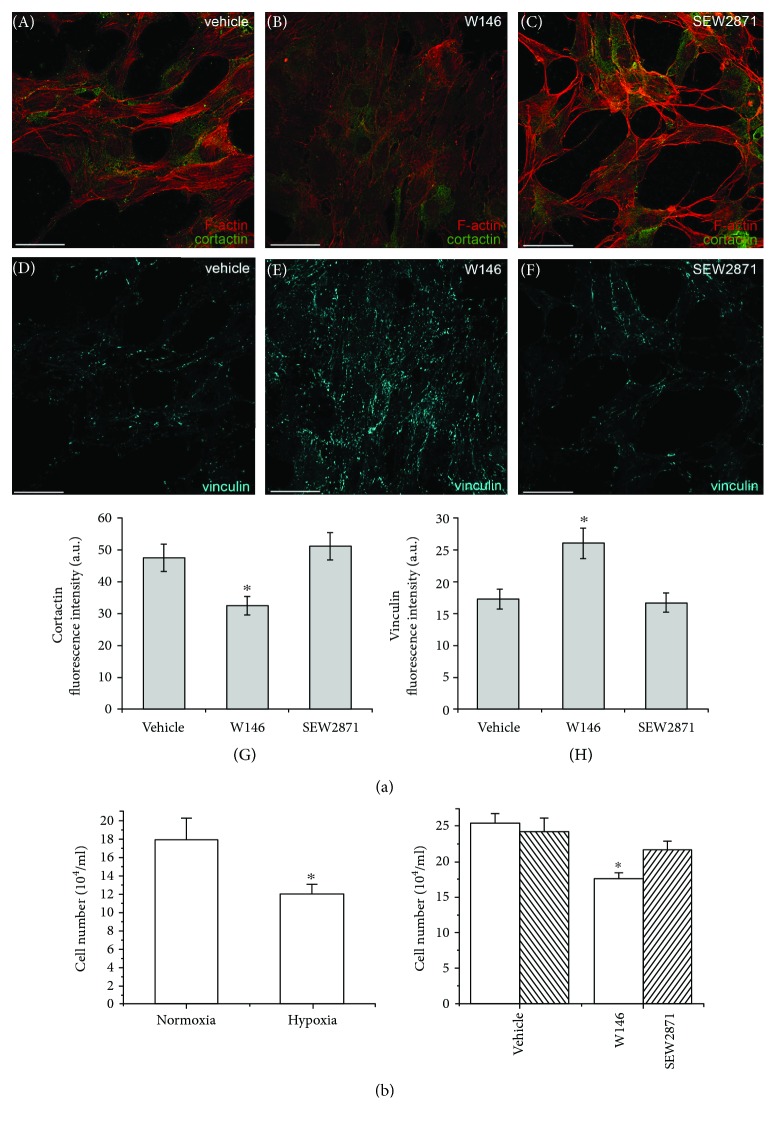
Cytoskeleton organization, cortactin and vinculin expression, and cell proliferation in BM-MSCs cultured under hypoxic conditions. (a, A–F) Representative immunofluorescence confocal images of BM-MSCs cultured on glass coverslips for 48 h under hypoxic conditions in the absence (vehicle) or in the presence of 2 *μ*M S1PR1 receptor antagonist, W146, or 2 *μ*M S1PR1 receptor agonist, SEW2871, fixed and stained (A–C) with Alexa 568-phalloidin to detect actin filaments (red) and immunostained with antibodies against cortactin (green) and (D–F) with antibodies against vinculin (cyan). Scale bar 50 *μ*m. The images are representative of at least three independent experiments with similar results. (a, G and H) Densitometric analyses of the intensity of the (G) cortactin and (H) vinculin fluorescence signals performed on digitized images. Data are mean ± S.E.M. (b) Cell proliferation analysis by cell counting. Synchronized BM-MSCs were incubated in growth medium for 48 h in normoxia and hypoxia (histogram on left) or in hypoxia in the absence (vehicle) or in presence of 2 *μ*M S1PR1 receptor antagonist, W146, or 2 *μ*M S1PR1 receptor agonist, SEW2871 (histogram on the right). Cells were collected and counted by TALI Cytometer as reported in Section 2. Data are mean ± S.E.M. of four independent experiments performed in quadruplicate. Significance of differences in (a) (G and H, Student's *t*-test), ^∗^*p* < 0.05 versus vehicle; in (b) (Student's *t*-test), ^∗^*p* < 0.05 versus normoxia or vehicle.

The morphological analysis revealed that the cells in hypoxia appeared more elongated and did not present the plasma membrane-associated F-actin structures (Figure 7(a), A), as observed in normoxia (Figure 3). In addition, the vehicle- and SEW2871-treated cells showed well-organized F-actin filaments (stress fibers), spanning parallel to the length of the cells (Figure 7(a), A–C) and a weak staining of cortactin (Figure 7(a), A, C, and G) which, however, appears to be colocalized with cortical actin (overlap coefficient *r*: vehicle, 0.922 ± 0.064; SEW2871, 0.919 ± 0.064, *p* > 0.05 versus vehicle). By contrast, as compared to control cells, W146-treated cells under hypoxia displayed a reduction of stress fiber assembly and of the expression of cortactin (Figure 7(a), B and G; overlap coefficient *r*: vehicle, W146, 0.944 ± 0.066, *p* > 0.05 versus vehicle). Of note, these cells showed an upregulation of the expression levels of vinculin, a focal adhesion protein involved in cell/matrix connection [62] (Figure 7(a), D, F, and H), suggesting that W146 treatment is not toxic but that the inhibition of S1PR1 signalling might act as a cell fate determinant. Consistent with these morphological features, cell proliferation was reduced in hypoxia as compared to normoxia and, in the same experimental conditions, W146 treatment further decreased cell number (Figure 7(b)).

Altogether, these data suggest that S1PR1-mediated signalling in hypoxic conditions is required for HIF-1*α* and MMP-2 expression/activity, cortactin expression, and reduction of vinculin expression as well as stress fiber formation and cell proliferation.

## 4. Discussion

MMP-2 preferentially degrading collagen and gelatin plays an important role in BM-MSC migration [14, 46, 47, 63] and in cell proliferation [64–66]. It has been also reported that MMPs can rapidly act on several specific substrates inside the cells, such as cytoskeleton and cytoskeleton-associated proteins [8, 67–69]. Therefore, the identification of physiological regulators of the MMP-2 function is of great biological relevance in BM-MSC field and could offer clues to improve their therapeutic efficacy. In such a view, our data appear intriguing and of potential clinical interest. In fact, here, we demonstrate, for the first time, the trophic role exerted by the signalling pathways downstream to the activation of S1PR1 in the modulation of the MMP-2 gelatinolytic activity and substantial cytoskeleton remodelling. In fact, we found that cells in normoxic conditions treated with S1PR1 antagonist W146 showed a reduction of MMP-2 expression/activity as well as of F-actin assembly and the disappearance of plasma membrane-associated F-actin structures, such as lamellipodia-like structures, filopodia, and small punctate structures (microspikes) associated with a decrease of cortactin expression, similarly to the cells treated with a specific MMP inhibitor. In addition, under hypoxic conditions the cells with blocked S1PR1 showed an upregulation of vinculin.

The observed lack of effects of S1PR1 agonist SEW2871 may suggest that the signalling pathways, mediated by S1PR1 and leading to MMP-2 activation, actin cytoskeleton reorganization and ECM remodelling, are already maximally activated by the presence of either endogenously released S1P. These data contribute to further suggest that the signalling pathway downstream of S1PR1 is constitutively activated by the endogenously formed and released S1P and it is a trophic response; in fact, only a downregulation of the receptor is able to perturb the BM-MSC response.

The involvement of other S1P receptor subtypes in mediating ECM remodelling, as reported in other studies, cannot be excluded [70–72]. Accordingly, in this study, we demonstrated that exoS1P is able to upregulate MMP-2 expression and promote actin assembly. Experiments are ongoing in our lab to deeply analyse the involvement of the other receptor subtypes. However, our preliminary results indicate that S1PR2 antagonist is not effective in regulating MMP-2 activity in BM-MSCs, whereas a specific antagonist of S1PR3 is not commercially available.

The relationship between MMP inhibition and reduction of cytoskeleton assembly [8] and the ability of the S1P signalling to regulate MMP-2 [17, 34, 35, 48, 49] and ECM remodelling [73, 74] are consistent with previous reports in different cell types. Moreover, our findings are also in accordance with the observation that the S1P/S1PR1 axis is able to stimulate, through the activation of Cdc42/Rac pathway, the translocation of cortactin towards the cell periphery, where the cortical actin contributes to form lamellipodia [75]. Thus, it can be supposed that S1P/S1PR1/MMP-2 system may have a similar function in BM-MSCs. On the other hand, since it has been demonstrated that cortactin is an essential regulator of MMPs [76, 77], we can also speculate that S1PR1 signalling may modulate MMP-2 expression/activity via regulation of cortactin expression.

A further intriguing result of this study is the autocrine action of S1P released by BM-MSCs via S1PR1. In fact, both the inhibition of SphK activity and the blockade of S1PR1 strongly affect ECM remodelling and MMP-2 activation.

The novelty and the importance of these findings rely on the fact that the autocrine/trophic role of the endogenously formed and released bioactive lipid S1P would make BM-MSCs less dependent on a niche created by other cells types underlying also the relevance of a specific S1PR subtype in such action.

Furthermore, our data represent the first evidence of a change in the expression level of S1P receptor subtypes in stem cells with a predominance of S1PR1 expression in high-density BM-MSC culture. These data may contribute to further suggest the potentiality of MSCs to modulate their own constitutive responses by this molecular mechanism, independently from extracellular signals.

High- and low-density BM-MSC culture can be considered to reflect the behaviour of BM-MSCs in vivo; indeed, it appears conceivable that BM-MSCs, which are rarefied in vivo (low density) in a normal/physiological tissue, display a higher degree of aggregation (high density) in a damaged tissue. It has been reported that many biological activities of MSCs could be influenced by the confluence of the cells [78]. For instance, a density-dependent behaviour of MSCs has been reported in the study by Lapi et al. [79], showing that the addition of hEGF to MSC culture is more efficient in promoting self-renewal in low-density versus high-density culture, indicating the requirement of specific signalling pathways in the two different conditions. In addition, it shall be considered that, in order to contribute to tissue repair, the cells need to be protected either against differentiation signals and apoptosis as well as to undergo proliferation before reaching the injury sites where they accumulate. After that, they presumably could switch cell program, stopping the self-renewal and the proliferation program and starting the commitment. Therefore, the novel finding of a significant increase in the content of S1PR1 in high-density culture might indicate a potential role of S1PR1-mediated signalling in the downregulation of self-renewal ability and/or, likely, promotion of cell proliferation/commitment. Further investigations are ongoing in our laboratory to address this topic. However, our reported finding that S1PR1 antagonist reduces cell proliferation and promotes cell growth arrest, but not the expression of apoptotic or autophagic markers, indicates the role of S1PR1-mediated signalling in the control of BM-MSC cell cycle.

Moreover, our findings may support that S1PR1 signalling can modulate cell proliferation via MMP-2 and cytoskeleton remodelling in BM-MSCs. In fact, it has been reported that (i) MMP-2 expression regulates MSC proliferation [64–66]; (ii) the actin cytoskeleton structure determines cell shape and the cytoskeleton-dependent cell morphology affects cell proliferation, in particular, actin-rich plasma membrane-associated structures, such as filopodia and small punctate structures (microspikes), are suggestive of cell spreading and that cells unable to spread show a reduced proliferation [57–60]; and (iii) cortactin, beside its role as a molecular scaffold for the formation of dynamic cortical actin-associated structures, has been shown to modulate specific molecular signalling targets involved in cell proliferation (e.g., ERK1/2) [56, 80]. Therefore, it is possible to speculate that the increase in S1PR1 expression in high-density BM-MSC culture is needed to prompt cortactin expression/MMP-2 activity and, likely, cell proliferation ability, by inducing changes in cell morphology and cytoskeletal organization.

In this study, we also found that S1PR1-mediated signalling plays a crucial role in BM-MSCs cultured under hypoxic conditions which resemble the microenvironment of damaged/repairing tissue. Moreover, since in literature hypoxia reduces the proliferative potential of BM-MSCs concomitantly to an enhancement of cell differentiation [81], our findings show that the S1PR1 antagonist reduces HIF expression may support the role of S1PR1 in the regulation of HIF-1*α*. In addition, since the stabilization of HIF-1*α* leads to cell cycle arrest in response to hypoxia in various cell types [82], the regulation of the expression of this transcriptional factor and its nuclear localization support the role of S1PR1-mediated signalling also in cell proliferation regulation under hypoxia. Many studies on the biologic activity of BM-MSCs have produced contradictory results regarding up- or downregulation of proliferation or differentiation of BM-MSCs [83–85] as well as the expression of MMP-2 in MSCs in hypoxia [86]; this could be dependent on the differences in the hypoxic conditioning protocols among the various studies, making comparison difficult. In any case, many of the cellular responses to reduced oxygen availability are mediated by the transcriptional activity of HIF-1 [61] and, accordingly, our results showed a significant increase in the expression level of this factor in the cells under hypoxia.

Our data concerning proliferation are in agreement with recent findings establishing a transcription-independent mechanism by which the stabilization of HIF-1*α* leads to cell cycle arrest in response to hypoxia in various cell types [82]. Of note, the finding reported in this study that the S1PR1 antagonist reduces HIF-1*α* expression and its nuclear localization suggests that S1PR1-mediated signalling could be required to maintain the expression/activity of HIF-1*α* under hypoxia, which contributes in supporting the previous findings of the role for S1P as a regulator of this transcriptional factor [87]. Interestingly, the S1PR1/HIF-1*α* system may be required to regulate F-actin assembly into stress fibers that we observed in the cells under hypoxia. It has been, in fact, demonstrated that HIF-1 regulates cytoskeletal reorganization, consisting mainly in stress fiber formation in different cell types by activating RhoA [88] as it has been shown also for S1P [35, 89]. Therefore, considering that RhoA negatively affects MMP-2 [90], we can speculate that S1PR1-mediated signalling may be important to maintain HIF-1*α* function which, in turn, upregulates RhoA; this event could downregulate MMP-2 and promote the formation of stress fibers. The appearance of stress fibers in hypoxia, concomitantly with the disappearance of the plasma membrane-associated F-actin structures, changes in cell shape from polygonal to elongated, and the reduction of cell proliferation were suggestive of an enhanced cell adhesion.

Finally, the upregulation of vinculin observed under hypoxia in the presence of the S1PR1 antagonist may suggest the role for S1PR1-mediated signal in contributing to downregulate vinculin which, besides its role as a force-sensitive adhesion protein, has been proved to be able to modulate the differentiation of BM-MSCs [62].

## 5. Conclusion

**Figure 8:**
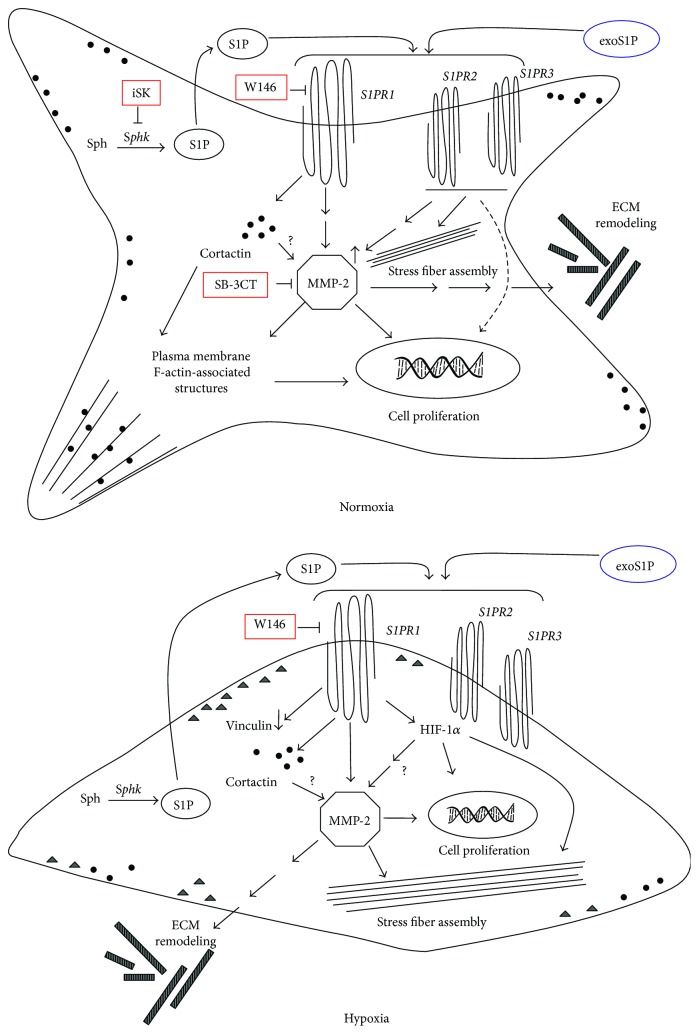
Schematic drawing summarizing the main conclusions of the study.

In this study, we demonstrated for the first time the trophic constitutive role exerted by the signalling pathways downstream of S1PR1 in maintaining the ability of BM-MSCs to modulate MMP-2 expression/activity, important for remodelling the surrounding ECM and reorganizing actin cytoskeleton in order to regulate cell proliferation (Figure 8).

Our findings may open new windows for a smart targeting of S1PR1 for the application in cell therapy, by preserving BM-MSC properties and potentiating their efficacy for tissue repairing purposes.
